# Acupuncture for irritable bowel syndrome comorbid with anxiety and depression: study protocol for a placebo run-in, randomized clinical trial

**DOI:** 10.3389/fpsyt.2026.1782020

**Published:** 2026-04-22

**Authors:** Xuezhou Wang, Weijuan Gang, Xiaoyan Wang, Hang Zhou, Jin Huo, Yingying Wang, Rongjun Li, Lingyu Qi

**Affiliations:** 1 The Institute of Acupuncture and Moxibustion, China Academy of Chinese Medical Sciences, Beijing, China; 2 International Acupuncture and Moxibustion Innovation Institute, School of Acupuncture-Moxibustion and Tuina, Beijing University of Chinese Medicine, Beijing, China

**Keywords:** acupuncture, irritable bowel syndrome, mental health, protocol, randomized controlled trial

## Abstract

**Background:**

Gastrointestinal and psychological symptoms jointly motivate patients with irritable bowel syndrome (IBS) to seek medical care, with the latter often exacerbating the difficulty of achieving overall improvement. Although acupuncture has been shown to improve gastrointestinal symptoms in the general IBS population, the evidence in individuals with psychological disturbances remains insufficient.

**Methods:**

This will be a placebo run-in, randomized controlled trial. After the 1-week sham acupuncture intervention run-in period, the 80 eligible IBS patients without strong placebo responses will be randomly assigned to the true acupuncture (TA) group and sham acupuncture (SA) group. During the 4-week intervention period, all patients will receive the assigned intervention three times per week, followed by an 8-week follow-up. The primary endpoint is the response rate, defined as the proportion of patients with a decrease of greater than or equal to 50 points in the IBS Symptom Severity Scale (IBS-SSS) score from baseline at week 4. Secondary outcomes include the response rates at other time points, original IBS-SSS scores, Hospital Anxiety and Depression Scale (HADS), Visual Analog Scale (VAS) for abdominal pain, satisfaction with bowel habits, blinding assessment, and Credibility/Expectancy Questionnaire. Safety will be monitored and recorded during the trial.

**Discussion:**

This trial will provide individualized evidence for addressing IBS with key comorbidities while excluding placebo response. The results of this trial will be published in a peer-reviewed journal.

**Clinical Trial Registration:**

https://itmctr.ccebtcm.org.cn/mgt/project/view/1985616257155727360, identifier ITMCTR2025002100.

## 1 Introduction

Irritable bowel syndrome (IBS) is a common disorder of gut-brain interaction characterized by abdominal pain associated with changes in stool form or bowel habits (1), affecting approximately 5–10% of the general population (2). Due to its recurrent and chronic nature, IBS significantly impairs quality of life and increases healthcare burden (3, 4). The annual direct and indirect costs of IBS are estimated to be about $10 billion across different countries and regions (5–7).

Beyond gastrointestinal symptoms, IBS is frequently associated with mental disorders (8). A recent meta-analysis revealed that the prevalence of anxiety and depression among patients with IBS was 39% and 29%, respectively, and that 23% of patients suffered from both conditions (9). Mental disorders in patients with IBS require increased attention, as they may aggravate disease severity and are associated with serious outcome such as disability or suicide (10). Current first-line pharmacological therapies, including laxatives, antidiarrhoeals, and antispasmodics, primarily target gastrointestinal symptoms and provide limited direct benefit for mental health (11). Due to safety concerns with long-term treatment, non-pharmacological approaches are receiving increasing attention.

Acupuncture has long been used as an option for the management of gastrointestinal symptoms (12), as well as primary psychological disorders (13, 14). Due to gut–brain interactions, patients with accompanying psychological symptoms may find it more difficult to achieve overall improvement. Our previous studies in the general IBS population have revealed that acupuncture is both safe and effective for alleviating gastrointestinal symptoms (15, 16). Although acupuncture appeared to improve mental health, no statistically significant difference was observed compared with the sham acupuncture. This unclear effect may be attributed to the mild baseline mental symptoms, and the absence of specific acupoints with sedative effects, both of which may have influenced outcomes. Therefore, to address the lack of evidence on this common comorbidity, the present study aims to evaluate the efficacy and safety of acupuncture in patients with IBS and coexisting mental health conditions.

## 2 Methods and analysis

### 2.1 Study design

**Figure 1:**
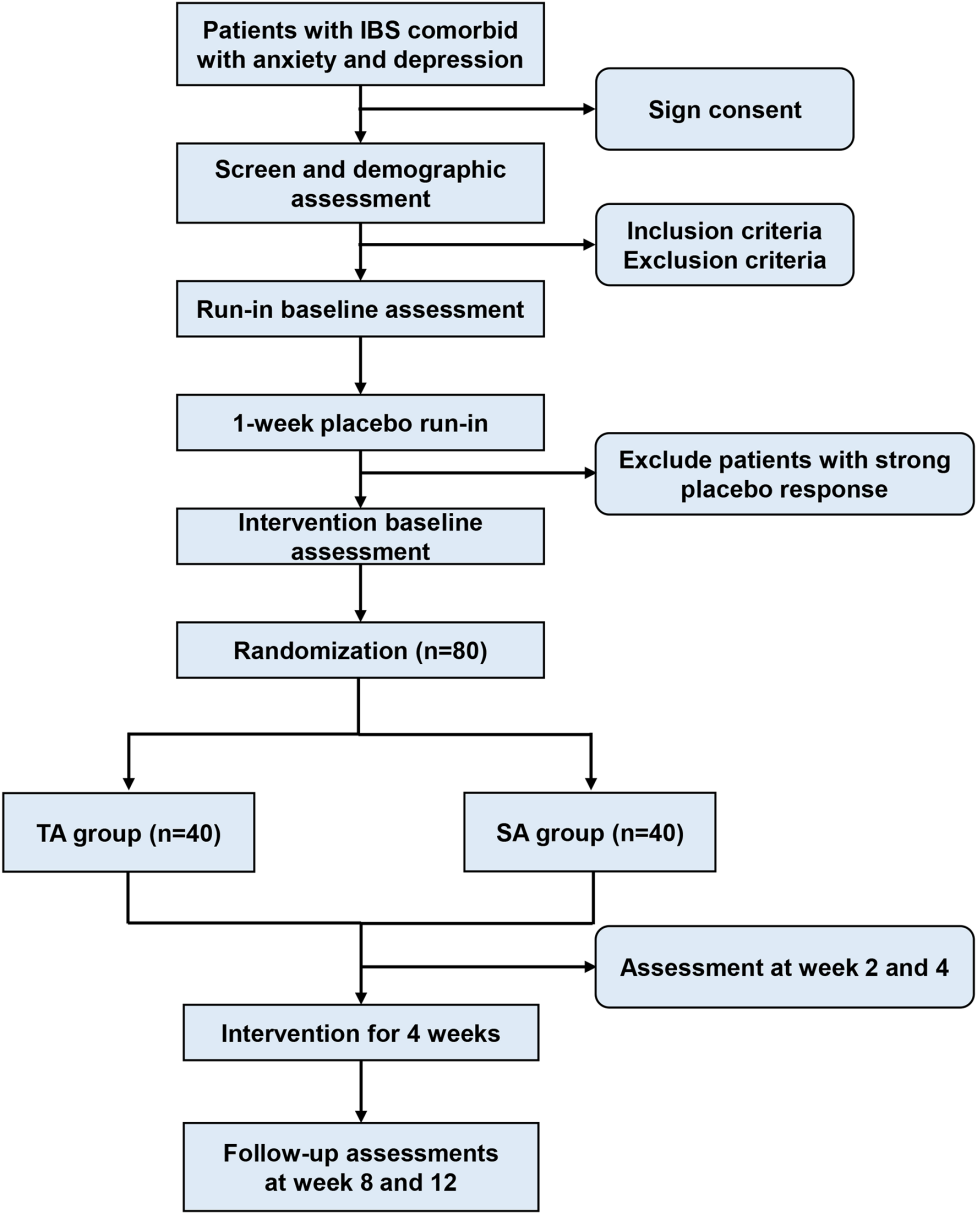
Study flowchart. IBS, irritable bowel syndrome.

This will be a randomized controlled clinical trial involving patients with IBS. Eligible patients will be randomly allocated to the true acupuncture group (TA) or the sham acupuncture group (SA) in a 1:1 ratio. The 13-week study consists of a 1-week placebo run-in period, a 4-week treatment period and an 8-week follow-up. The study protocol was approved by the Medical Ethics Committee of Xiyuan Hospital, China Academy of Chinese Medical Sciences (Approval ID: 2025XLW019-1), and was registered (International Traditional Medicine Clinical Trial Registry: ITMCTR2025002100). The present trial will be performed following SPIRIT 2025 statement (Additional file 1). **Figure 1** shows the study flowchart. All personnel involved in this trial will receive standardized training on the trial procedures.

### 2.2 Participants

#### 2.2.1 Diagnostic criteria

According to the 2016 Rome IV diagnostic criteria (17), IBS is diagnosed by the presence of recurrent abdominal pain, on average at least one day per week in the last three months, accompanied by two or more of the following criteria:

- Related to defecation;
- Associated with a change in frequency of stool;
- Associated with a change in form (appearance) of stool.

Criteria fulfilled for the last 3 months with symptom onset at least 6 months before diagnosis.

#### 2.2.2 Inclusion criteria

- Individuals aged between 18 and 75, irrespective of gender;
- Participants fulfill the diagnostic criteria of Rome IV for irritable bowel syndrome;
- The Irritable Bowel Syndrome Symptom Severity Scale (IBS-SSS) scores ≥75 points, and the Hospital Anxiety and Depression Scale (HADS) scores ≥11 points;
- No suicidal behavior within the past 3 months and the Suicidal Behaviors Questionnaire-Revised (SBQ-R) scores <7;
- Prospective participants do not have undergone acupuncture treatment for irritable bowel syndrome or taken psychiatric medications within the preceding six months;
- Should a participant be on medications that alleviate irritable bowel syndrome symptoms, such as traditional Chinese medicine, Chinese patent medicine, antidiarrheal drugs, antispasmodics, intestinal antibiotics, or probiotic preparations, prior to the commencement of treatment, they must discontinue use at least two weeks prior to the onset of treatment.

#### 2.2.3 Exclusion criteria

- History of organic intestinal disorders, including inflammatory bowel disease, microscopic colitis, celiac disease, or Crohn’s disease;
- Diabetes mellitus and thyroid dysfunctions;
- Severe acute or chronic organic diseases, kidney or liver diseases;
- History of abdominal surgeries (including appendectomy, hemorrhoidectomy, and polypectomy conducted more than three months prior);
- Pregnancy or lactation;
- History of alcohol or drug misuse;
- Patients currently participating in other clinical trials.

### 2.3 Recruitment and informed consent

Patients will be recruited at the Acupuncture Hospital of the China Academy of Chinese Medical Sciences through print or online advertisements, including newspapers, websites, and hospital posters. Prior to formal participation, the investigator shall inform the patient of the trial’s background, study procedures, and the study’s significance. Comprehensive information regarding the potential benefits, risks, and discomforts associated with participating in the study will be furnished to guarantee that the patient thoroughly understands the study. Patients will decide whether or not to participate in the trial and may withdraw at any time during the trial. For those patients who refuse to participate in this trial, they will be advised of other conventional treatments if requested. All relevant personal information about the patient, which may include occupation, medical history, condition, physical examination and laboratory results, will be kept confidential.

In addition to the detailed informed notification process, the following efforts will be made to ensure patient adherence: First, the scientific background of the research team and recent advances in disease research will be introduced. Second, the operation, clinical efficacy, safety, and possible mechanisms of acupuncture will be explained. Third, patients who complete the trial will receive an additional free acupuncture treatment at the end of the study.

### 2.4 Run-in period

Eligible patients will first undergo a one-week placebo run-in period, designed to identify those with a strong placebo response and exclude them prior to randomization. This approach allows for a more accurate assessment of the net effect of the intervention and enables participants with poor compliance to voluntarily withdraw before randomization (18, 19). For acupuncture, excluding strong placebo responders facilitates a clearer evaluation of its specific effects, ensuring that observed improvements in gastrointestinal and psychological symptoms can be more confidently attributed to the intervention rather than placebo responses. After run-in baseline assessments, all patients will receive a single-blind sham acupuncture intervention three times per week for one week, following the same procedure as the SA group. Patients who achieve a reduction of ≥50 points on the IBS-SSS during this period will be considered placebo responders and excluded from randomization.

### 2.5 Randomization and allocation concealment

Patients who are not excluded after the run-in period will undergo treatment baseline assessments and be randomly assigned in a 1:1 ratio to the TA or SA group. An independent statistician, not involved in the trial’s implementation or statistical analysis, will generate the randomization sequence using SAS 9.3 software. Sequentially numbered, opaque, sealed envelopes will be used to implement the random allocation. Recruiters will register participants, while acupuncturists will assign the interventions.

#### 2.5.1 Blinding

Due to the characteristics of acupuncture treatment process, only the acupuncturist and their assistant will have access to the randomization information. Patients, clinical recruiters, outcome assessors, data managers, and statisticians will be blinded to the group allocation. Unblinding of the study data will occur after the database has been locked and all participants have completed the trial.

Permitted circumstances for unblinding include (1): when a patient experiences a serious adverse event necessitating knowledge of the assigned intervention for clinical management (2); when the trial protocol contains major design flaws that threaten the scientific integrity of the results or constitute a hazard to participant rights and safety; and (3) legal requirements. If unblinding is necessary, the principal investigator will be immediately notified, and an assessment panel will evaluate the need for unblinding. If it is deemed necessary, the custodian responsible for safeguarding the randomization information will release the details according to established protocols, with the unblinding process carefully documented. The ethics committee will be informed upon completion of the unblinding.

### 2.6 Interventions

The acupuncture treatment protocol is based on the principles of Traditional Chinese Medicine and informed by literature review and expert consensus. Only licensed acupuncturists with over 5 years of clinical experience, will perform the interventions. Eligible patients will be randomized to receive either TA or SA intervention during the 4-week treatment phase, with three treatments per week (ideally every other day). Patients will lie flat on a treatment bed, and single-use sterile needles (0.30 mm diameter, 40 mm length; Hwato, Suzhou, China) will be applied.

#### 2.6.1 TA group

**Figure 2:**
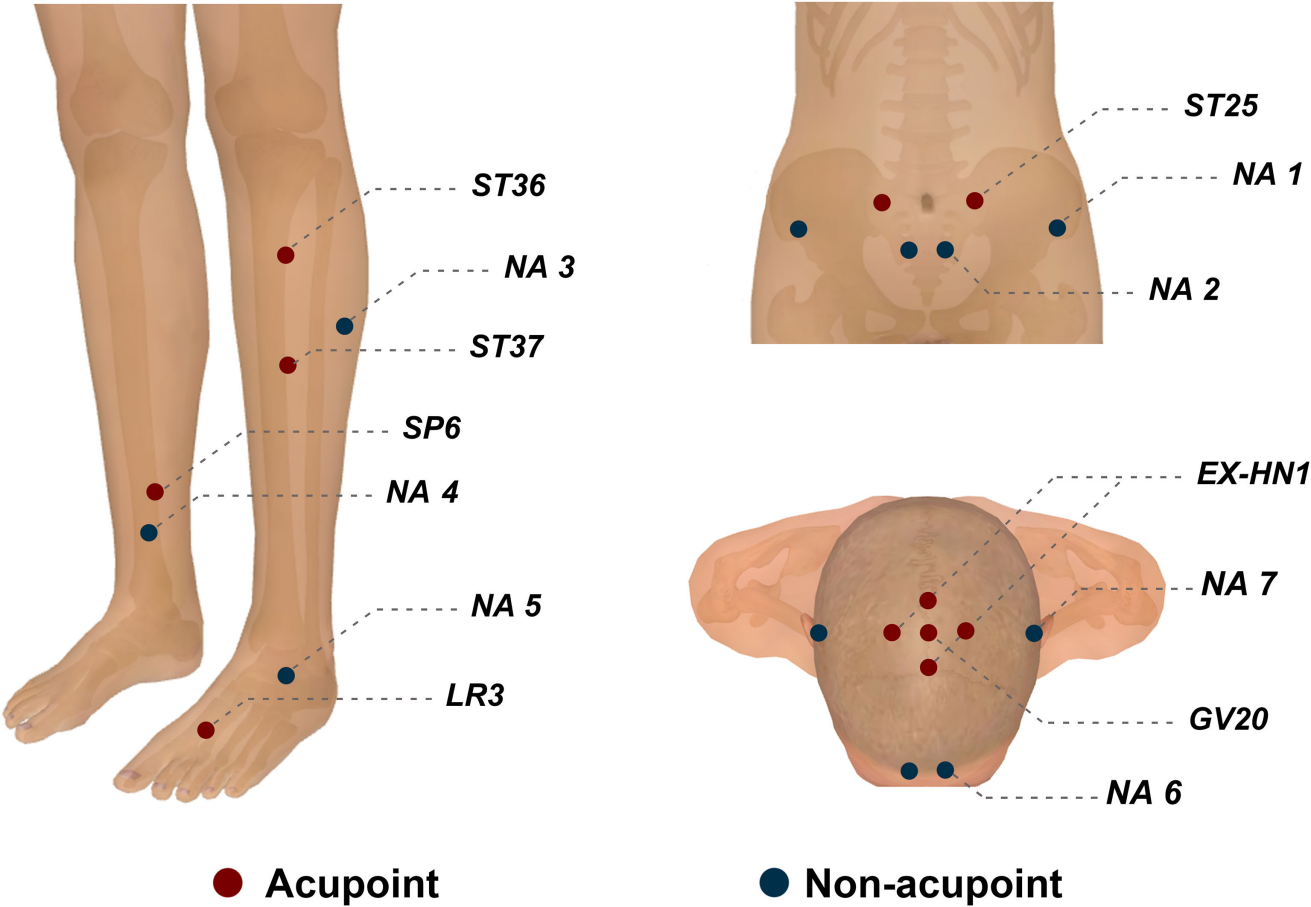
Acupoint location. NA, non-acupoints.

Seven fixed acupoints will be used for the TA group (**Table 1**; **Figure 2**). *Baihui* (GV20) and *Sishencong* (EX-HN1) will target negative emotions (e.g., anxiety, depression), while *Tianshu* (ST25), *Zusanli* (ST36), *Shangjuxu* (ST37), *Sanyinjiao* (SP6), and *Taichong* (LR3) will address gastrointestinal symptoms. For abdominal and lower limb acupoints, the acupuncturist will insert needles into deep tissue layers. Following insertion, manipulations of twirling, lifting, and thrusting will be performed to achieve deqi (a sensation of soreness, numbness, distension, and heaviness) after sterilization. The needles will be retained for 30 minutes, with the deqi manipulation performed every 10 minutes during retention.

**Table 1:** Locations of acupoints for acupuncture intervention.

Acupoint	Location
Baihui (GV20)	On the head, 5 cun^a^ directly above the midpoint of the anterior hairline
Sishencong (EX-HN1)	On the top of the head, 1 cun lateral to the Baihui acupoint in the front, back, left, and right directions, totaling 4 acupoints
Tianshu (ST25)	On the horizontal line of the navel, 2 cun beside the anterior midline
Zusanli (ST36)	3 cun directly below ST35, and one finger-breadth lateral to the anterior border of the tibia
Shangjuxu (ST37)	On the anterolateral aspect of the leg, 6 cun inferior to the ST35, and one finger-breadth lateral to the anterior border of the tibia
Taichong (LR3)	In the depression anterior to the junction of the first and second metatarsal bones
Sanyinjiao (SP6)	On the tibial aspect of the leg, posterior to the medial border of the tibia, 3 cun superior to the prominence of the medial malleolus

^a^ 1 cun (≈20 mm) is defined as the width of the interphalangeal joint of the participant’s thumb.

#### 2.6.2 SA group

For the SA group, 7 non-acupoints (NA) away from meridians and conventional acupoints will be used (**Table 2**; **Figure 2**). Superficial skin penetration (2–3 mm) will be performed at these points. Patients in the SA group will receive the same treatment duration as those in the TA group, with the deqi manipulation replaced by pretended manipulation every 10 minutes.

**Table 2:** Locations of acupoints for sham acupuncture intervention.

Non-acupoint	Location
Non-acupoint 1	On the abdomen, 2 cun^a^ superior to anterior superior iliac spine, between the gallbladder meridian and the spleen meridian
Non-acupoint 2	On the abdomen, 2 cun inferior to navel, 1 cun beside the anterior midline, between the kidney meridian and the stomach meridian
Non-acupoint 3	On the lateral aspect of the leg, 3 cun inferior to GB34, between gallbladder meridian and bladder meridian
Non-acupoint 4	On the leg, 2 cun superior to the medial malleolus, in the middle of the medial tibia, between the liver meridian and the spleen meridian
Non-acupoint 5	On the leg, the midpoint of the line between GB40 and ST41, between the gallbladder meridian and the stomach meridian
Non-acupoint 6	On the head, 0.5 cun lateral to the midpoint of the anterior hairline
Non-acupoint 7	On the head, at the midpoint of the line connecting GB8 and TE20, between the gallbladder meridian and the triple energizer meridian.

^a^ 1 cun (≈20 mm) is defined as the width of the interphalangeal joint of the participant’s thumb.

#### 2.6.3 Rescue medicine

Participants will be instructed to maintain their usual diet and lifestyle throughout the study. When necessary, loperamide or polyethylene glycol 4000 will be permitted as rescue medication under the individualized guidance of a gastroenterologist. Furthermore, psychiatric specialists will be arranged to assess whether additional treatment is required based on patients’ scale scores and disease status, not limited to pharmacotherapy. Any use of rescue medication or other concomitant treatments will be documented, including measure, time and dosage.

### 2.7 Outcomes

#### 2.7.1 Primary outcome

The primary outcome will be the response rate, defined as the proportion of patients achieving a reduction of ≥50 points in the IBS-SSS (20) from baseline, which is recommended by the U.S. Food and Drug Administration (21). The primary endpoint will be the between-group comparison of response rate at week 4. The IBS-SSS consists of five items rated on a visual analog scale (0–100): severity of abdominal pain, frequency of abdominal pain, severity of abdominal distension, dissatisfaction with bowel habits, and interference with quality of life. Each item contributes equally to the total score, with an overall range of 0–500.

#### 2.7.2 Secondary outcomes

Original IBS-SSS scores will be assessed as secondary outcomes at baseline and at weeks 2, 4, 8, and 12. The response rate will also be evaluated as a secondary outcome at baseline and at weeks 2, 8, and 12.

Hospital Anxiety and Depression Scale (HADS) (22). Symptoms of anxiety and depression will be assessed using the HADS at baseline and at week 2, 4, 8, and 12. The HADS is a validated 14-item instrument comprising two subscales for anxiety and depression. Scores are interpreted as follows: 0–7, no symptoms; 8–10, suspected symptoms; and 11–21, definite symptoms (23).

Visual Analog Scale (VAS) (24). The worst abdominal pain over the preceding 24 hours will be evaluated by VAS (0–10), with 0 indicating no pain and 10 indicating the worst imaginable pain. VAS will be evaluated at baseline and at week 2, 4, 8, and 12.

Satisfaction with bowel habits (25). Patients’ satisfaction with defecation will be assessed using a 7-point Likert scale, with response options ranging from “strongly disagree” to “strongly agree.” This scale provides an appropriate balance between sensitivity and usability, allowing for more nuanced assessment than a five-point scale while avoiding the cognitive burden of scales with more response options. Assessments will be conducted at baseline and at week 2, 4, 8, and 12.

Credibility/Expectancy Questionnaire (26). The credibility and expectancy to the intervention will be measured within 5 minutes after the first intervention session completed.

Blinding. The blinding assessment will be conducted by asking participants to guess their intervention allocation at week 2 and 4.

Safety. All adverse events occurring during the study will be monitored and recorded, regardless of their presumed relationship to the intervention. Adverse events will be classified according to severity and causality. Mild adverse events are those that do not substantially interfere with daily activities and require no specific treatment. Moderate adverse events involve noticeable discomfort or functional impairment and may require intervention. Severe adverse events are defined as life-threatening events, events resulting in significant disability, or those requiring hospitalization. For moderate adverse events, the decision to continue participation will be made in accordance with the patient’s wishes. Participants experiencing serious adverse events will be withdrawn from the trial and managed by the relevant clinical departments.

### 2.8 Data management

Paper-based materials will serve as the primary medium for data collection and storage. Clinical recruiters will be responsible for printing and archiving recruitment materials, case report forms, and informed consent documents. Acupuncturists and assistants will maintain treatment records and adverse event logs, which will be securely stored throughout the trial. To preserve blinding, research data will remain non-referential until all trial data have been collected and submitted to the principal investigator.

A standardized case report form will be established prior to enrollment, and data will be entered into EpiData software. Outcome assessors will record outcome data in the corresponding case report form sections. Any data modifications must be documented with reasons, timestamps, and signatures. Participant privacy will be strictly protected. Upon trial completion, data will undergo double entry and verification before database locking.

#### 2.9 Quality control

Study quality will be ensured through protocol development, standardized training, and data verification. Experts in acupuncture, gastroenterology, and statistics participated in protocol development. All personnel will receive unified training before trial initiation. During the trial, investigators will maintain appropriate communication with participants to support adherence and data integrity. Data quality checks will focus on missing values, outliers, and inconsistencies. Data entry, coding, and analysis will follow unified standards. Final data review and database locking will be conducted before unblinding.

#### 2.10 Sample size

There is a lack of direct evidence on acupuncture for patients with IBS comorbid with anxiety and depression. The response rate for the SA group was derived from studies in general IBS populations because sham effects are expected to be minimal and not substantially influenced by comorbid anxiety or depression, and based on our previous study it was approximately 25% (15). For the TA group, both the established effects of acupuncture in general IBS populations and evidence from large-scale meta-analysis showing moderate-to-large effect size (Cohen’s d = 0.66) of acupuncture for depression were taken into account (13). Considering the potential secondary improvement in gastrointestinal symptoms via acupuncture targeting anxiolytic and sedative acupoints, we estimated a response rate of approximately 60% in the TA group by the end of the week 4. With α = 0.05, 36 participants per group are required to achieve 80% power. Accounting for a 10% dropout rate, a total of 80 participants will be recruited.

#### 2.11 Statistical analysis

The following hypothesis will be tested simultaneously for the primary outcome:

H0: There is no difference in the response rate between the TA and SA group.

H1: There is a significant difference in the response rate between the TA and SA group.

Analyses of the treatment efficacy will be based on the modified intention-to-treat (mITT) population, which consists of all participants who were randomized and had baseline data. The safety analysis will be carried out on the safety population, which comprises individuals who received at least one dose of treatment after randomization with full recorded safety outcome. The incidence of adverse events will be determined by dividing the number of patients who experienced at least one adverse event by the total number of patients. Missing data in outcomes will be imputed five times by multiple imputation method, with the mean values been used for analysis. Continuous data will be presented as mean and standard deviation or as median and interquartile range, while categorical data will be presented in frequency and proportion. For response rate, comparison will be performed using chi - square test. For other endpoints, continuous variables will be compared using T test or the Wilcoxon rank sum test, and categorical variables will be examined using the chi - square test or Fisher’s exact test. To assess the robustness of the findings, sensitivity analyses will be conducted by truncating the data at the first use of rescue medication in repeated-measures models, and by including rescue medication and demographic characteristics use as covariates to control for its potential impact on the treatment effect estimate. Subgroup analyses will be conduct on patients with different subtypes of IBS (IBS with diarrhea, IBS with constipation, IBS with mixed bowel habits, and IBS unclassified) to evaluate the differences in the efficacy of acupuncture. All analysis will be two side and conducted using SAS 9.3 software, and a *P* value less than 0.05 will be considered statistically significant.

## 3 Discussion

Acupuncture is widely used in clinical practice across China for the management of gastrointestinal disorders. For functional gastrointestinal conditions such as IBS, acupuncture may offer a distinctive advantage by its capacity to regulate both physiological and psychological processes, thereby enabling simultaneous improvement of emotional disturbances and gastrointestinal symptoms (27). This characteristic mitigates the limitation inherent to pharmacotherapy, which may address symptoms without involving the abuse of psychotropic medications. Nevertheless, current international clinical guidelines do not strongly recommend acupuncture for IBS, largely due to a lack of high-quality clinical evidence (28). Therefore, building on our previous series of studies (15, 16), we consider it necessary to further explore more individualized evidence, with the aim of providing additional therapeutic options for this patient population.

Relative to our prior studies, this protocol incorporates key optimizations concerning acupoint selection rationale and control group design. The *Huangdi Neijing*, an ancient Chinese medical canon, stipulates that “the principles of acupuncture must always be rooted in the spirit”. Accordingly, we selected *Baihui* (GV20) and *Sish*encong (EX-HN1) to modulate patients’ negative affective states (29). For the remaining acupoints targeting IBS symptoms, their selection has been previously justified and validated in our prior study (16). Mechanistic research has substantiated that electroacupuncture at these points may modulate murine brain function via suppression of neuroinflammation. Furthermore, this trial employs non-acupoint superficial needling as the control intervention. Previous research utilizing sham acupuncture devices assessed the placebo effect of acupuncture and confirmed its specific therapeutic efficacy for IBS. Subsequent investigations necessitate isolating the specific physiological effects of acupuncture; thus, non-acupoint shallow needling is deemed a more appropriate control. Another important optimization concerns the selection of the primary outcome (30). Although both our previous studies and the current trial used response rate as the primary endpoint, the definitions differ. In our earlier work, composite responders were defined by improvements in abdominal pain and diarrhea frequency only. In contrast, the present study uses the IBS-SSS, which reflects the overall disease burden. Responders are defined as patients achieving a reduction of at least 50 points in IBS-SSS score from baseline. This definition is consistent with U.S. Food and Drug Administration recommendations for IBS clinical trials and allows a more comprehensive evaluation of both gastrointestinal symptoms and associated psychological status.

This study possesses several limitations. First, the inherent nature of acupuncture manipulation precludes practitioner blinding. Second, being a single-center RCT, patient recruitment sources are relatively homogenous, potentially limiting broad representativeness. However, recruitment advertisements will be disseminated across multiple hospital departments and diverse media platforms to enhance patient diversity. Third, although the placebo run-in period effectively excludes strong placebo responders, it may increase recruitment difficulty and prolong the trial duration.

In conclusion, this clinical trial protocol is designed to evaluate the efficacy and safety of a treatment strategy integrating spirit-regulation and spleen-fortification via acupuncture for IBS through an RCT. The implementation process is expected to adhere to standardized protocols, yielding scientifically robust data. The findings are anticipated to generate evidence-based medical data supporting the clinical application of acupuncture for IBS, establish a foundation for subsequent research, and ultimately confer benefit upon patients.

## 4 Ethics and Dissemination

The study protocol has received approval from the Medical Ethics Committee of Xiyuan Hospital of China Academy of Chinese Medical Sciences. Any important protocol modifications will be approved by the ethics committee. The randomized controlled trial has acquired the registration number (ITMCTR2025002100) and will be carried out in line with the regulations of the Declaration of Helsinki. The trial results will be published in a peer-reviewed journal.
